# Echocardiographic and hemodynamic changes in patients with high-grade varicocele

**DOI:** 10.17305/bb.2022.8854

**Published:** 2023-05-01

**Authors:** Batuhan Ergani, Azmi Eyiol, Mustafa Karabiçak, Mesut Gitmez, Muslu Kazım Körez

**Affiliations:** 1 Department of Urology, Beyhekim Training and Research Hospital, Konya, Turkey; 2 Department of Cardiology, Beyhekim Training and Research Hospital, Konya, Turkey; 3 Department of Urology, Batman Regional State Hospital, Batman, Turkey; 4 Department of Cardiology, Batman Regional State Hospital, Batman, Turkey; 5 Department of Biostatistics, Selçuk University Faculty of Medicine, Konya, Turkey

**Keywords:** Echocardiography, high grade varicocele, semen analysis, spermatic vein diameter

## Abstract

Varicocele is abnormal tortuosity and dilatation of the pampiniform plexus veins within the spermatic cord. Varicocele is associated with testicular atrophy, hypogonadism, impaired semen analysis values, or decreased testosterone production. Varicocele is a progressive disease and should be treated because it may be a systemic disease that can be associated with cardiovascular abnormalities. We hypothesize in this study that cardiovascular and hemodynamic pathologies may occur in varicocele patients. In this prospective, multicentric, multidisciplinary study, patients diagnosed with high-grade left varicocele in the urology clinic underwent semen analysis, total testosterone determination, and scrotal Doppler ultrasonography. In addition, blood pressure measurement and echocardiographic evaluation were performed by blinded cardiologists in both the varicocele patients and the healthy control group. The study was carried out with 103 varicocele patients and 133 healthy individuals who formed the control group. Diastolic blood pressure (*P* ═ 0.016), left ventricular end diastolic (*P* < 0.001) and systolic diameter (*P* < 0.001), ejection fraction (*P* < 0.001), pulmonary arterial pressure (*P* < 0.001), and aortic distensibility (*P* < 0.001) values were significantly higher in varicocele patients compared with controls; interventricular septum wall thickness (*P* ═ 0.022), aortic systolic (*P* < 0.001) and diastolic diameter (*P* < 0.001), aortic systolic (*P* < 0.001) and diastolic diameter index (*P* < 0.001), and aortic stiffness index (*P* < 0.001) values were significantly lower in varicocele patients. The mean aortic distensibility of non-normozoospermic group was lower than that of normozoospermic group (*P* ═ 0.041). There was no statistically significant relationship between thickest vein diameter in spermatic cord and cardiological parameters. This study showed that symptomatic patients with high-grade varicocele had a higher risk of cardiovascular and hemodynamic disease. We recommend that men with high-grade symptomatic varicocele with impaired semen analysis undergo cardiovascular and hemodynamic evaluation regardless of their spermatic vein diameter.

## Introduction

Varicocele is abnormal tortuosity and dilatation of the pampiniform plexus veins within the spermatic cord. Varicocele is seen in approximately 15% of men and is one of the most important causes of male infertility, and up to 40% of men with primary infertility have varicoceles [1]. Varicocele might cause testicular atrophy, scrotal pain, hypogonadism, increased gonadotropins, impaired semen analysis values, or decreased testosterone production [2]. Although the pathophysiology of varicocele is not clear yet, a combination of the following conditions contributes to the symptoms seen in men with varicocele: impaired valvular mechanisms, increased oxidative stress, increased scrotal temperature and pressure, and toxic metabolites accumulation [3].

Varicocele is a progressive disease, and some high-grade varicocele should be treated after diagnosis to prevent further decline in impaired testicular functions [4]. Some indications for high-grade varicocele to be treated include bilateral varicocele (if bilateral clinical or left clinical right subclinical), symptomatic varicocele, testicular atrophy, and impaired spermatogenesis [1]. Different opinions also recommend varicocelectomy for low testosterone levels, also known as hypogonadism. This is because there is a link between varicocele and decreased testosterone [5]. Moreover, hypogonadism is associated with metabolic risk factors, such as obesity, hyperglycemia, dyslipidemia, and insulin resistance, with cardiovascular diseases, such as hypertension, and with increased mortality due to all causes [6].

Vascular tonus is under the combined effect of vasoconstrictor and vasodilator mediators. Vascular tonus and valvular mechanisms impaired by imbalances between these vasoactive mediators may contribute to pathological events, such as vasospasm, hypertension, and varicose veins [7]. In fact, high-grade varicocele was associated with endothelial dysfunction and increased vasoconstriction [8]. In another retrospective study, the probability of developing cardiovascular disease in men with varicocele was found to be higher than in those who underwent varicocelectomy [9].

All this limited information suggests that varicocele may be a systemic disease that can coexist with cardiovascular abnormalities. We hypothesize that cardiac and vascular pathologies may occur in varicocele patients. For this purpose, this study aimed to investigate the relationship between the semen analysis results, pampiniform plexus vein diameter, and cardiovascular and echocardiographic parameters between healthy individuals and high-grade varicocele patients. According to the results of our study, early diagnosis and treatment of possible vascular and cardiac pathologies in newly diagnosed high-grade varicocele patients and their complications will be possible.

## Materials and methods

### Patient selection

This study was carried out prospectively, multicentric and multidisciplinary in two different urology and two different cardiology clinics between November 2021 and August 2022. Male patients in the 18–50 age group with high-grade and left varicocele who applied to the urology outpatient clinic were included in the study to form the “patient” group. The control group consisted of healthy volunteer men in the 18–50 age group without any known disease. Men with the following conditions were not included: those with a history of testicular surgery or varicocelectomy, those with low and moderate varicocele, those with only right or bilateral varicocele, those with pituitary insufficiency, those with a known cardiological disease, those with known kidney or liver insufficiency, those with known thyroid disease, those with diabetes or dyslipidemia, those using exogenous androgen or gonad stimulating preparations, and professional athletes.

Physical examination, the gold standard in the diagnosis of varicocele, was performed on patients in the urology outpatient clinic whose symptoms and complications (infertility, testicular pain and/or atrophy, and palpable lesion) resulted in suspicion of varicocele. Varicocele was graded according to Dubin and Amelar classification: low-grade (palpable only during the Valsalva maneuver), moderate-grade (tangible without the Valsalva maneuver), or high-grade (visible without the need for palpation) [1]. Accordingly, semen analysis, total testosterone, and scrotal Doppler ultrasonography at supine position were requested from the patients whose physical examination revealed a high-grade varicocele in the left testicle. Subsequently, their blood pressure (BP) measurements and echocardiography were performed in the cardiology clinic. On healthy volunteers in the control group, only BP measurement and echocardiography were performed. The age, body mass index (BMI; weight in kilograms divided by the square of the height in meters), and smoking status of all participants were recorded. Sperm parameters were grouped as normozoospermia, oligozoospermia, asthenozoospermia, teratozoospermia, and azoospermia according to the values recommended by the World Health Organization (WHO) [10].

### Echocardiographic analysis

All patients underwent standard transthoracic echocardiography in the left lateral position by two blinded cardiologists in two cardiology clinics. Two-dimensional and M-mode echocardiographic parameters were obtained using an echocardiography device (Philips S3-1, Germany/Esaote MyLab 7, Italy) with a 2–4 MHz transducer. M-mode measurements were made according to the criteria recommended by the American Society of Echocardiography [11]. BP measurements were performed on the right and left arm of the subjects in the sitting position by a blind, trained observer who works in echocardiography room. BP measurement was done twice, 5 min apart. Systolic BP and diastolic BP values were obtained with a mercury sphygmomanometer in the first and fifth Korotkoff phases. The average of four BP measurements was taken into the analysis. The difference between systolic BP and diastolic BP was pulse pressure. Hypertension was when the mean systolic BP ≥ 140 mmHg and mean diastolic BP ≥ 90 mmHg [12]. Then, echocardiography measured the following parameters: left ventricular-end diastolic and systolic diameters, right ventricular diameter, atrial diameters, aortic diameter, ejection fraction, interventricular septum wall thickness and posterior wall thickness, pulmonary artery pressure, and aortic systolic and diastolic diameters. Aortic systolic and diastolic diameter indices and aortic distensibility and aortic stiffness index were calculated using the echocardiographic and hemodynamic data. The following formulas were used:

aortic systolic diameter index ═ aortic systolic diameter/body surface area;

aortic diastolic diameter index ═ aortic diastolic diameter/body surface area;

aortic stiffness index ═ ln(systolic BP/diastolic BP)/[(systolic aortic diameter-diastolic aortic diameter)/diastolic aortic diameter];

aortic distensibility ═ 2×(systolic aortic diameter-diastolic aortic diameter)/[(diastolic aortic diameter)×(PP)] [13].

### Ethical statement

The study protocol was reviewed and approved by the Batman Regional State Hospital Institutional Review Board (approval number: 08.10.2021/275). Informed consent was obtained by all participants when they were enrolled. The procedures followed were in accordance with the Helsinki Declaration.

**Table 1:** Demographical and cardiologic parameters of the groups

	**Study groups**		
**Variable**	**Varicocele (*n* ═ 103)**	**Control (*n* ═ 133)**	***P* value**
*Demographical characteristics*			
Age (years), median (range)	24 (18–49)	25 (18–47)	0.568^1^
Body mass index (kg/m^2^)	24.81 ± 2.61	22.52 ± 1.45	<0.001^2^
Smoking	66 (64.1%)	22 (16.5%)	<0.001^3^
*Semen analysis*			
Normozoospermia	56 (54.4%)		
Oligozoospermia	32 (31.1%)		
Asthenozoospermia	36 (35%)		
Teratozoospermia	23 (22.3%)		
Azoospermia	2 (1.9%)		
Low testosterone level	4 (3.8%)		
Thickest vein diameter in spermatic cord (mm)	3.49 ± 0.85		
*Cardiological parameters*			
Systolic blood pressure (mm/Hg)	107 (106–110)	106 (102–112)	0.100^1^
Diastolic blood pressure (mm/Hg)	69.76 ± 5.59	67.98 ± 5.50	0.016^4^
Pulse pressure (mm/Hg)	38.63 ± 5.06	40.11 ± 6.65	0.055^2^
Left ventricular end diastolic diameter (mm)	45.80 ± 2.95	44.06 ± 1.71	<0.001^2^
Left ventricular end systolic diameter (mm)	28.57 ± 1.71	27.68 ± 0.95	<0.001^2^
Left atrium diameter (mm)	30.50 ± 1.95	30.27 ± 1.87	0.370^4^
Aortic diameter (mm)	23.83 ± 2.04	24.15 ± 1.64	0.176^4^
Ejection fraction (%)	65 (60–65)	60 (60–65)	<0.001^1^
Interventricular septum wall thickness (mm)	9.89 ± 0.46	10.02 ± 0.30	0.022^2^
Posterior wall thickness (mm)	8.93 ± 0.45	9.02 ± 0.29	0.076^2^
Right atrium diameter (mm)	25.1 (23.8–26.7)	24.8 (23.8–28.2)	0.786^1^
Right ventricular diameter (mm)	24.3 (23.2–26.2)	24.2 (23.2–27.3)	0.621^1^
Pulmonary arterial pressure (mm/Hg)	14 (10–17.5)	5 (5–12)	<0.001^1^
Aortic systolic diameter (mm)	24.6 (22.9–25.7)	26.1 (23.6–28.1)	<0.001^1^
Aortic diastolic diameter (mm)	20.1 (18.2–21.5)	22.2 (18.7–24.2)	<0.001^1^
Aortic systolic diameter index (mm/m^2^)	12.99 ± 1.29	14.68 ± 1.09	<0.001^4^
Aortic diastolic diameter index (mm/m^2^)	10.57 ± 1.22	12.27 ± 1.32	<0.001^4^
Aortic distensibility	1.2 (0.9–1.4)	0.8 (0.6–1.3)	<0.001^1^
Aortic stiffness index	1.88 (1.60–2.30)	2.61 (1.66–3.31)	<0.001^1^

Data are expressed as mean ± standard deviation or median with interquartile range (IQR) for numerical variables, as appropriate. For categorical variables, data are described as count (*n*) and percentage (%). ^1^Mann–Whitney U test. ^2^Aspin–Welch *t*-test. ^3^Pearson chi-square test. ^4^Independent samples *t*-test.

### Statistical analysis

Statistical analyses were performed using the R version 3.6.0 (The R Foundation for Statistical Computing, Vienna, Austria; https://www.r-project.org). Shapiro–Wilk’s test of normality and *Q*–*Q* plots were used to check the normality of the data. Levene’s test was used to assess the homogeneity of variances. Numerical variables were expressed as mean ± standard deviation, median with range, or median with interquartile range (IQR). Categorical variables were expressed as number (*n*) and percentage (%). A Mann–Whitney U test, Alpin–Welch’s *t*-test, Pearson chi-square test, and independent sample *t*-test were run to determine the statistical significance difference between varicocele and control groups according to demographical characteristics, semen analysis, hormonal pathology, and cardiological parameters. Besides, we conducted the Mann–Whitney U test, Alpin–Welch’s *t*-test, and independent sample *t*-test to compare the cardiological parameters in terms of semen analysis groups. Also, the relationship between cardiological parameters and thickest vein diameter in spermatic cord was examined via Spearman’s rho, Kendall’s Tau B, and Pearson correlation analysis. A *P* value less than 0.05 was considered to be statistically significant.

## Results

### General data

Three hundred patients were examined for inclusion in our study. The study was carried out with a total of 236 participants, with 103 varicocele patients and 133 healthy individuals forming the control group after the exclusion criteria were applied. The average age of the participants was 26.7 years (range 18–49); mean BMI was 23.51 kg/m^2^. A total of 88 (37.2%) participants were smokers. Testosterone levels were low in 4 (3.8%) varicocele patients. Of these patients with varicocele, 56 (54.4%) had normozoospermia, 5 (4.8%) had teratozoospermia, 2 had (1.9%) asthenozoospermia, 4 had (3.8%) oligoteratozoospermia, 6 had (5.8%) asthenoteratozoospermia, 20 (19.4%) had oligoasthenozoospermia, 8 (7.7%) had oligoasthenozoospermia, and 2 (1.9%) had azoospermia. Reflux flow was present in the scrotal doppler ultrasonography in all varicocele patients.

**Figure 1.**
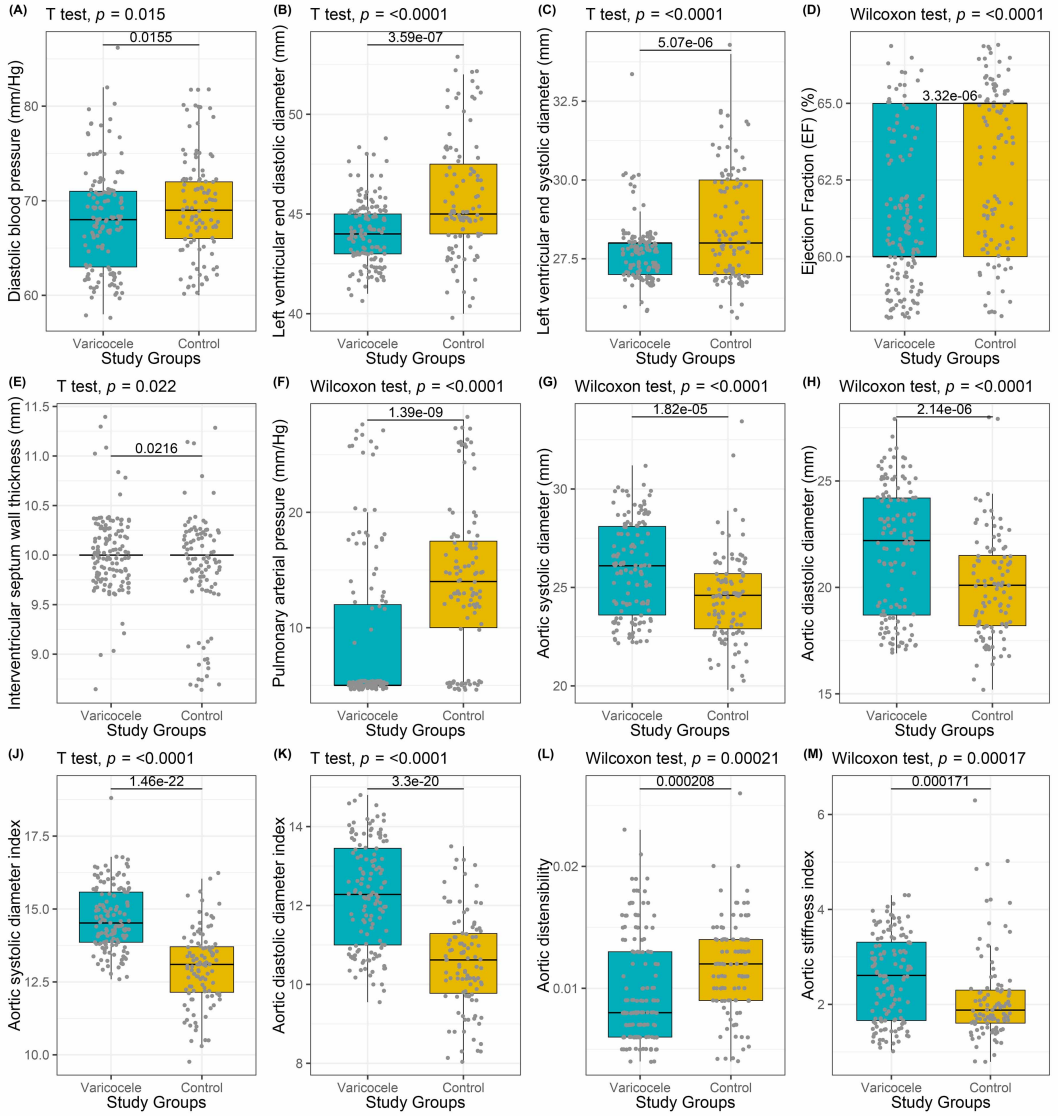
Cardiological and hemodynamic parameters found to be significant between the groups.

### Demographic and cardiological parameters between groups

The median age was 24 (range 18–49) in varicocele patients and the median age was 25 (range 18–47) in the control group. The age distribution was similar between the groups (*P* ═ 0.568). The mean BMI (24.81 ± 2.61 vs 22.52 ± 1.45; *P* < 0.001) and smoking frequency (*n* ═ 66 [64.1%] vs *n* ═ 22 [16.5%]; *P* < 0.001) of varicocele patients was higher than that of the control group. According to the semen analysis, of the 103 varicocele patients had one or more: 56 (54.4%) had normozoospermia, 32 (31.1%) had oligozoospermia, 36 (35%) had asthenozoospermia, 23 (22.3%) had teratozoospermia, and 2 (1.9%) had azoospermia. The mean value of the thickest vein diameter in spermatic cord was 3.49 ± 0.85 mm (range 2.1–6.4) in varicocele patients. Diastolic BP, left ventricular end diastolic diameter, left ventricular end systolic diameter, ejection fraction, pulmonary arterial pressure, and aortic distensibility values in varicocele patients were significantly higher compared to controls, whereas the interventricular septum wall thickness, aortic systolic diameter, aortic diastolic diameter, aortic systolic diameter index, aortic diastolic diameter index, and aortic stiffness index value were lower in varicocele patients. There was no statistically significant difference between varicocele and control groups in terms of systolic BP, pulse pressure, left atrium diameter, aortic diameter, posterior wall thickness, right atrium diameter, and right ventricular diameter. The comparison of both groups is shown in Table 1, and the parameters with significant results are additionally shown in Figure 1.

### Cardiological results by semen analysis

The mean aortic distensibility of non-normozoospermic group was lower than that of normozoospermic group (1.24 ± 0.41 vs 1.08 ± 0.34; *P* ═ 0.041). No statistical significance was found in other evaluated parameters. The comparison of cardiological parameters according to semen analysis is shown in Table 2.

**Table 2:** The comparison of cardiological parameters according to semen analysis

	**Semen analysis**		
**Cardiological parameters**	**Non-normozoospermia (*n* ═ 47)**	**Normozoospermia (*n* ═ 56)**	***P* value**
Systolic blood pressure (mm/Hg)	108 (106–110.5)	107 (105.75–110)	0.402^1^
Diastolic blood pressure (mm/Hg)	69.72 ± 5.53	69.79 ± 5.69	0.955^2^
Pulse pressure (mm/Hg)	40 (37–42)	39 (36–40.25)	0.114^1^
Left ventricular end diastolic diameter (mm)	46.19 ± 3.17	45.46 ± 2.74	0.214^2^
Left ventricular end systolic diameter (mm)	28.89 ± 1.84	28.30 ± 1.56	0.082^2^
Left atrium diameter (mm)	30.89 ± 2.00	20.16 ± 1.86	0.057^2^
Aortic diameter (mm)	24.13 ± 2.18	23.57 ± 1.90	0.169^2^
Ejection fraction (%)	65 (60–65)	65 (60–65)	0.433^1^
Interventricular septum wall thickness (mm)	9.89 ± 0.48	9.89 ± 0.45	0.993^2^
Posterior wall thickness (mm)	8.96 ± 0.46	8.91 ± 0.44	0.601^2^
Right atrium diameter (mm)	25.50 ± 2.51	25.43 ± 2.47	0.879^2^
Right ventricular diameter (mm)	25.13 ± 2.30	24.78 ± 2.81	0.507^2^
Pulmonary arterial pressure (mm/Hg)	15 (12–17)	13.5 (5–18)	0.619^1^
Aortic systolic diameter (mm)	24.74 ± 2.40	24.31 ± 2.05	0.329^2^
Aortic diastolic diameter (mm)	20.33 ± 2.43	19.73 ± 2.38	0.207^2^
Aortic systolic diameter index (mm/m^2^)	13.07 ± 1.39	12.93 ± 1.21	0.585^2^
Aortic diastolic diameter index (mm/m^2^)	10.68 ± 1.20	10.48 ± 1.23	0.397^2^
Aortic distensibility (×10^2^)	1.08 ± 0.34	1.24 ± 0.41	0.041^2^
Aortic stiffness index	2.02 (1.68–2.33)	1.77 (1.47–2.15)	0.067^1^

Data are expressed as mean ± standard deviation or median with interquartile range (IQR) for numerical variables, as appropriate. ^1^Mann–Whitney U test. ^2^Independent samples *t*-test. ^3^Aspin–Welch *t*-test.

### Cardiological results by spermatic vein diameter

There was no statistically significant relationship between the thickest vein diameter in spermatic cord and cardiological parameters (all *P* values > 0.05). The correlation analysis between the thickest vein diameter in spermatic cord and cardiological parameters is shown in Table 3.

**Table 3:** The correlation analysis between thickest vein diameter in spermatic cord and cardiological parameters

	**Thickest vein diameter in spermatic cord (mm)**	
	**Correlation**	
**Cardiological parameters**	**coefficient**	***P* value**
Systolic blood pressure (mm/Hg)	−0.014	0.888^1^
Diastolic blood pressure (mm/Hg)	−0.029	0.772^1^
Pulse pressure (mm/Hg)	0.111	0.264^2^
Left ventricular end diastolic diameter (mm)	0.173	0.080^1^
Left ventricular end systolic diameter (mm)	0.176	0.075^1^
Left atrium diameter (mm)	0.061	0.544^2^
Aortic diameter (mm)	0.177	0.073^2^
Ejection fraction (%)	0.173	0.081^1^
Interventricular septum wall thickness (mm)	−0.091	0.260^3^
Posterior wall thickness (mm)	−0.064	0.429^3^
Right atrium diameter (mm)	0.101	0.311^1^
Right ventricular diameter (mm)	0.031	0.758^1^
Pulmonary arterial pressure (mm/Hg)	0.083	0.406^1^
Aortic systolic diameter (mm)	0.095	0.338^1^
Aortic diastolic diameter (mm)	−0.048	0.629^1^
Aortic systolic diameter index (mm/m^2^)	0.188	0.057^2^
Aortic diastolic diameter index (mm/m^2^)	0.069	0.489^1^
Aortic distensibility (×10^2^)	0.119	0.233^1^
Aortic stiffness index	−0.179	0.071^1^

^1^Spearman’s *rho*. ^2^Pearson. ^3^Kendall’s Tau B.

## Discussion

Varicocele is associated with many conditions that may impair human health. For example, there is a relationship between varicocele and low testosterone levels [5]. According to the literature, low testosterone levels increase cardiovascular comorbidities, such as myocardial infarction, angina, or coronary heart disease [14]. Although it was determined that our patients had a higher cardiac and hemodynamic risk compared to the healthy population, only four patients had low testosterone levels.

In a varicocele study examining functional and morphological changes, α1-adrenoceptor agonist sensitivity was higher in dilated spermatic veins. This increase was directly proportional to the degree of varicocele. The vascular cGMP level is responsible for the vasorelaxant effect of nitric oxide. It was lower in high-grade varicoceles [8]. A similar increase in susceptibility to α1-adrenoceptor agonists was also shown in hypertension models in other studies [15]. Increased venous pressure and hypertension in spermatic veins cause varicocele formation. Although not evaluated in our study and, therefore, it is assumed that there is a dysfunction in the valves based on the literature. This pathophysiological similarity between increased venous pressure and hypertension in spermatic veins was detected in our patients. Then, we concluded that while diastolic BP was statistically significantly higher in varicocele patients, systolic BP was also higher than in the control group.

Varicocele is associated with high levels of oxidative stress. While the effects of reactive oxygen radicals may impair spermatogenesis, they may also have systemic effects. Indeed, a high level of oxidative stress is associated with cardiovascular risk [16]. Inflammation plays an important role in coronary artery ectasia and peripheral varices, and there may be an indirect relationship between these two conditions [17]. This relationship can be explained by endothelial inflammation caused by reactive oxygen radicals. The negative effects of varicocele on cardiological parameters of our patients may be due to the systemic effect of endothelial inflammation caused by reactive oxygen radicals.

A study conducted in a fertility clinic investigated the relationship between spermiogram results and medical comorbidities of men with semen analysis. They reported a significant relationship between cardiovascular disease (hypertension, peripheral vascular disease, cerebrovascular diseases, and nonischemic heart diseases) and semen abnormalities [18]. Similar to the literature, some cardiovascular and echocardiographic abnormalities detected significantly lower/higher in our patients without normozoospermia.

Aortic stiffness is a parameter of the biomechanical properties of the aorta. It reflects the aortic distensibility, aortic elasticity, and mechanical strength of the aortic wall. Changes in aortic stiffness are related to various diseases, such as coronary artery disease. Since varicocele may result from a systemic disease associated with common cardiovascular abnormalities, there may be an indirect relationship between aortic stiffness and varicocele. In a study of 77 patients, although the result was not statistically significant, aortic stiffness in varicocele patients was lower than that in the control group [19]. In our study, we found that the aortic stiffness index in varicocele patients was statistically significantly lower, in parallel with the literature.

There is no study in the literature evaluating cardiovascular and hemodynamic parameters according to the diameter of the spermatic vein. According to the criteria used in the study by Sincer et al., varicocele grading was performed using scrotal Doppler ultrasonography. The presence of reflux lasting more than 1 s after the Valsalva maneuver, regardless of the high-grade (grade 3) varicocele vessel diameter, was defined as grade 0, <2 mm; grade 1, 2–3 mm; and grade 2, 3–5 mm. There were no significant differences in cardiological or hemodynamic parameters between high-grade varicocele patients and the control group [19, 20]. In this sense, our study is the first in the literature. We concluded that spermatic vein diameter has no significant effect on cardiological or hemodynamic parameters.

Since varicocele reduces fertility potential, it is necessary to analyze this disease not only from an organic perspective but also from a psychosomatic perspective. One study investigated the relationship between the risk of cardiovascular mortality and fatherhood over a 10.2 years follow-up period. The risk of cardiovascular mortality increased after the age of 50 in men who could not have a child compared to men with more than one child [21]. Another study stated that infertile men had a higher risk of developing ischemic heart disease over the years [22]. In light of this information, considering varicocele patients who apply with infertility, obtaining the opinion of cardiologists is relevant to reduce the risk of mortality of the patients with negative cardiovascular and hemodynamic parameters.

### Limitations

In the following, the limitations of this study are pointed out. First, in the examination of cardiological parameters according to semen analysis, no comparison could be made with pure subgroup analyses due to the insufficient number of patients according to other sperm parameters (oligozoospermia, asthenozoospermia, and teratozoospermia or combinations thereof) except for normozoospermia. Therefore, which impaired sperm parameter was associated with which cardiological or hemodynamic disorder is unknown. For such an interpretation, studies involving a very high number of patients are needed. Second, since patients are not followed up for a long time, the relationship between the duration of symptoms and mortality of cardiological disorders is unknown. Third, significantly higher BMI and smoking frequency in varicocele patients may have affected our results. Patients may be at risk because of smoking. Fourth, the study is prospective and includes some negative results, but a sample calculation was not made because we could not predict beforehand that negative results would be significant. Finally, we do not know the short- and long-term outcomes and the effects of varicocelectomy on the echocardiographic and hemodynamic results in younger and older patients with varicoceles. This idea may be the hypothesis of a future study.

## Conclusion

According to our results, high-grade varicocele is associated with significant cardiovascular and hemodynamic impairments compared to the normal population. With a significant decrease in aortic distensibility, we recommend that men with high-grade varicocele in the non-normozoospermic group should undergo cardiovascular and hemodynamic evaluation regardless of their spermatic vein diameter. With the advantage of early diagnosis, a follow-up medical or invasive treatment modality can be applied if necessary following a cardiological examination.
